# Mercury Pollution, Treatment and Solutions in Spent Fluorescent Lamps in Mainland China

**DOI:** 10.3390/ijerph15122766

**Published:** 2018-12-06

**Authors:** Zhongguo Li, Puqi Jia, Fu Zhao, Yikun Kang

**Affiliations:** 1 College of Earth and Environmental Sciences, Lanzhou University, Lanzhou 730000, China; jpq@lzu.edu.cn; 2 School of Mechanical Engineering, Purdue University, West Lafayette, IN 47907, USA; fzhao@purdue.edu; 3 Division of Environmental and Ecological Engineering, Purdue University, West Lafayette, IN 47907, USA; 4 Engineering Laboratory for Municipal Waste Pollution Control Technology and Equipment Research, Lanzhou 730000, China; qq332929861@163.com

**Keywords:** mercury pollution, treatment, fluorescent lamps, spent fluorescent lamps, LED, mainland China

## Abstract

With the increasing awareness of energy conservation and environmental protection, high energy-consuming incandescent lamps have been largely withdrawn from the stage of mainland China’s lighting industry because the main raw material for electricity production-coal-produces mercury pollution when burned and energy-saving fluorescent lamps have made considerable progress. However, fluorescent lamps emit mercury, which still causes environmental pollution. In this work, the existing problems in the development of fluorescent lamps, and in the collection and treatment of spent fluorescent lamps were analyzed. The contributions of various external factors to the above problems were evaluated based on fuzzy theory. Finally, solutions to control the pollution of mercury from fluorescent lamps and spent fluorescent lamps were proposed. Results show that the biggest problem that causes mercury pollution is the first among three factors: energy conservation and mercury emission from fluorescent lamps and spent fluorescent lamps, spent fluorescent lamp collection and spent fluorescent lamp treatment. The best way to solve these problems is by developing an energy-saving and environment-friendly light emitting diode (LED) industry in mainland China.

## 1. Introduction

In 1974, phosphors were first developed by Philips in the Netherlands. They emit red, green and blue lights which are sensitive to human’s eyes [1]. When applied in illumination, they can form triphosphor fluorescent lamps with advantages of high efficiency, good color and long lifetime, which makes fluorescent lamps widely used in various lighting places. This was an important milestone in the history of the development of the lighting industry.

Fluorescent lamps (FLs) typically consists of a phosphor-coated glass tube with electrodes at both ends. The lamp tubes are filled with an inert gas (usually argon) and a certain amount of mercury, partly in vapor form. The mercury vapor is excited when current is applied to the electrodes, and the ultraviolet radiation emitted by the excited mercury is incident on the phosphor and makes it excite visible light [2]. Mercury (Hg) is consumed in many ways during the lamp operation. For examples, mercury vapor can diffuse into the glass wall, be adsorbed on the surface of the phosphor coating, or be consumed at the cathode area [3].

Mercury, the most persistent hazardous pollutant in heavy metals, can migrate and transfer anywhere via flowing rivers and atmospheric circulation [4,5,6], continually accumulate in organisms [7], and even pollute the Earth’s biosphere by cycling in major environmental compartments and across national boundaries on a global scale for a long time [8,9]. It was reported that approximately 50% of mercury in the environment is from anthropogenic sources [10,11]. The transboundary movement of mercury among air, water and biota has caused global pollution [12]. The atmospheric transport of mercury is rapid and the time scale for global mixing of the troposphere is estimated to be only about 1 year, principally limited by the exchange of air between the Northern Hemisphere and the Southern Hemisphere [13]. Therefore, atmospheric mercury pollution is much more likely to cause transboundary harm through long-range environmental transport compared to release to land.

Fluorescent lamps can usually work for over 6000 h, but they eventually burn out once there is no enough mercury left in the vapor form. Thus, an adequate amount of mercury vapor is needed to secure a constant light output during the illumination process and achieve long lamp lifetimes [14,15]. Excessive mercury was added to the tube by most small manufacturers, although minimum amount of mercury was enough to maintain FL’s normal working. The evaporability of liquid mercury can cause very high mercury consumption, including the emission part, during the manufacture process of FLs. Therefore, it is difficult to accurately control the amount of mercury per lamp. Depending on the types of lamps and manufacturers, milligrams to tens of milligrams of mercury were added [16]. The small production scale of most lighting appliance manufacturers has made it difficult for the government to supervise the product quality of FLs during the production process. This is a big challenge for the dosage control of mercury in FLs.

Backward production technology is another reason for the excessive injection of mercury. Although China has introduced large numbers of FL production lines from abroad since the 1980s, considerable economic benefits promoted the participation of private enterprises, most of whom produced FLs by hand [17]. However, not only is the addition of the correct amount of mercury difficult to control, but also the impure gases left in the FL tube are difficult to remove in the manual process and these impure gases left in the FL tube are contaminants that cause mercury losses [18]. In addition, residual water brought by impure gases or solids will affect the work efficiency of mercury. Positive mercury ion forms stable compounds with water or its possible decomposition products (OH and H radicals). Although this kind of mercury reaction is reversible, it is slowly consumed and ultimately accounts for a considerable amount of mercury emissions [19].

In addition, over half of injected mercury is still left in spent fluorescent lamps (SFLs). If these SFLs cannot be safely treated, the mercury will be emitted into environment and cause tremendous harm to the environment and humans. The State Council of China issued the Notice of the State Council on Key Work in Building an Economical Society in the Near Future, especially in the next two years, as early as 2005 [20]. The contributions of mercury emission in China were unchanged, which were ranked as follows: coal combustion, metallurgical industry, cement and steel production, PVC production by calcium carbide, medical care (thermometer and sphygmomanometer), battery production, lamps from the period of 2000–2008 [21], and the contribution of mercury emissions from lamps was small. But the industrial patterns were tending to develop economically. A new pattern was bound to emerge and maybe make some changes on the contribution share of mercury emission. Therefore, it is necessary to understand which processes in the production, use, disposal and recycle of FLs cause the mercury pollution, find what methods are available to deal with mercury pollution, and confirm how to develop mercury-free lamps.

## 2. Methods

### 2.1. Research Methods

The pollution reasons and treatment suggestions of mercury in SFLs were researched by: (1) literature from Google Scholar and China National Knowledge Internet (CNKI) databases, (2) report summaries from OFweek, Arctic Monitoring, Assessment Programme and United Nations Environment Programme, and so on, and (3) government and institution websites, such as the Ministry of Industry and Information Technology of the China, the Ministry of Science and Technology of the China, the Ministry of Ecology and Environment of the China, European Commission, National Bureau of Statistics and the Metal Encyclopedia.

### 2.2. Evaluation Method

A comprehensive evaluation method based on fuzzy theory was employed in this paper. Fuzzy theory has been widely applied and plays an important role in emissions analysis in multiple fields [22]. Some air traffic control (ATC) system evaluations are difficult to accurately quantify because they may be fuzzy and stochastic. As a result, the theory of fuzzy set-valued statistics is more realistic to give a general description of a range. Fuzzy comprehensive evaluation was built on the basis of fuzzy transformation theory and maximum membership principle. In this study, factors related to evaluated events were considered, and then comprehensive evaluation on the pollution control situation of mercury from FLs and SFLs was decided.

*Step 1*: Determine the factor fields of the evaluated object

If there are *i* evaluation factors in the fields, they can be expressed as: *X* = {*x*_1_, *x*_2_, …, *x*_i_}.

*Step 2*: Determine the fields of level comment

If there are *j*-levels in the fields, they can be expressed as: *Y* = {*y*_1_, *y*_2_, …, *y*_j_}.

Each of the levels corresponds to a fuzzy set.

*Step 3*: Single-factor evaluation and fuzzy relation matrix

When the level and fuzzy sets were conducted, we quantify each factor *x_i_* and *y_j_* to evaluate the subject gradually. Then fuzzy relation matrix *μ_R_* can be drawn as: *μ_R_* (*x_i_*, *y_j_*) = *r_ij_*.

*Step 4*: Determine the indicator weight

The correlation between five factors (Economy, Government, Business, Public and Environment) and mercury pollution is fuzzy and stochastic. According to [22], fuzzy theory can establish a quantitative analysis on the base of provided qualitative data. An expert grading method was used to explain the correlation between mercury pollution and its influencing factors in this paper. We invited 30 experts to evaluate the correlation values of each factor which was assigned six experts. All experts specialized in the assigned research areas and have professional qualifications to give evaluating indicators. According to their understanding and knowledge on the actual situations of influencing factors and mercury pollution in Mainland China, they give the correlation values between mercury pollution and influence factors. The accuracy of the experts’ evaluating values is as reliable as that we know who is thin and tall in a group. Finally, the correlation value of each factor was obtained by calculating the mathematic mean of the six correlation values and is shown in Section 3.2.

## 3. Results and Discussion

### 3.1. Problems of FLs and SFLs

#### 3.1.1. Energy Conservation and Mercury Emission from FLs and SFLs

Energy supports social development and people’s lives. Human activities get the energy they need from energy resources such as fossil fuels (including coal), nuclear fuel, or renewable energy. During the burning of coal for producing electricity in China, 1 ton of coal produces about 3000 kW·h of electricity and releases 20–1950 mg of mercury. The content of mercury in most coals is in the range of 10–1000 mg/ton with an arithmetic average value of 150 mg/ton [23]. The luminous efficiency of a FL is about five times that of an incandescent lamp (IL). Therefore, FLs of the same luminous efficiency as ILBs (1 ton coal consumed) consumes 200 kg of coal, producing 4–390 mg of mercury. Consequently, the use of energy-saving FLs has significantly reduced the consumption of energy and the emission of mercury from coal-fired power plants, but a FL contains a few milligrams of mercury in its tube and several billion FLs are produced in China every year, resulting to up to tens of tons of mercury consumed [24].

According to the China Statistics Yearbook [25], 3429.5 million tons coal were consumed in mainland China in 2011. Consequently, 514.43 tons mercury were discharged from coal combustion based on the value of 150 mg mercury per ton coal, which makes coal the biggest mercury source. It is reported that 20.45 tons mercury were consumed in domestic FLs production in 2011 and most of it was discharged to the atmosphere [26]. Hence, we can learn the fact that the level of mercury consumed in FLs is about 4% of the mercury emissions from coal combustion. To save energy, the Chinese Government released the Roadmap to Gradually Phase out Incandescent Light Bulbs in Mainland China (Draft for Comments) in November 2011 [27]. Then, the usage of coal for thermal power would start to reduce. That is to say, the ratio of mercury consumed in FLs to mercury emissions from coal combustion would have gradually increased year by year.

Assuming 4 billion of lamps are lit for 8 h each day in mainland China, 58.4 million tons or 233.6 million tons of coal are needed when using the same luminous efficiency lamps: 15 W compact fluorescent lamps (CFLs) or 60 W ILBs. Then over 1.97 tons mercury (1.17 tons from coal-fired power and 0.8 tons from spent CFLs—one-quarter of CFLs) for CFLs based on the minimum quality of mercury (0.8 mg) were injected in one CFL and 4.67 tons mercury (from coal-fired power) for ILBs were discharged every year, respectively. In a word, although FLs are energy-saving and much less mercury is discharged, there is hope they can be replaced by some kind of more energy-saving and mercury-free lamps with the advanced manufacturing technology in the field of illumination.

China is the largest FL consuming and producing country in the world. In 2011, production of FLs in China was approximately 7 billion, amongst which the domestic production of CFLs was about 4.7 billion, accounting for over 80% of the world’s production [28]. According to the China’s national standard “Requirements for concentration limits for certain hazardous substances in lighting equipments (QB/T 2940-2008)”, the amount of mercury in FLs is limited to 10 mg, and that in CFLs is 5 mg [29]. Thus, besides the products for export, once this batch of 1.53 billion FLs and 1.9 billion CFLs cannot work anymore, maybe up to 15.3 tons mercury from SFLs and 9.5 tons mercury from SCFLs would be released into the environment if nothing is done in China.

**Figure 1:**
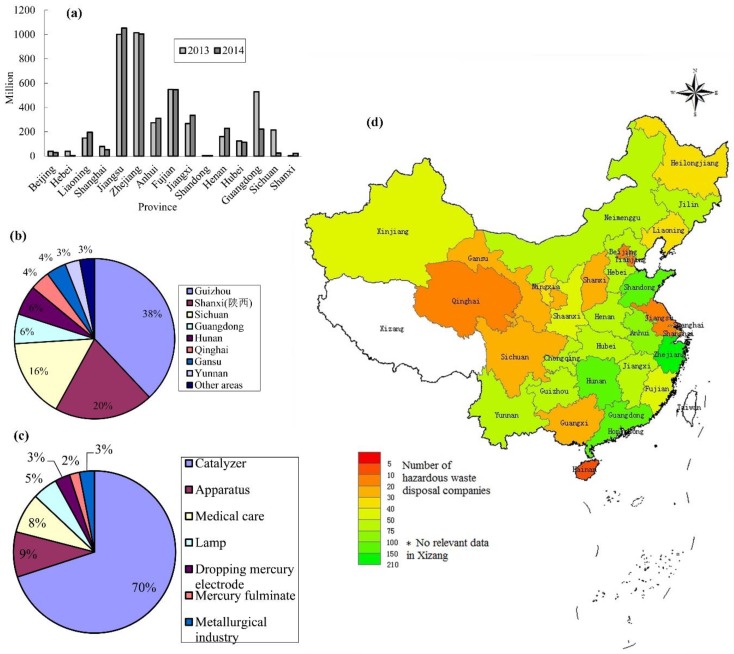
(**a**) Output of FLs in some provinces of China (data from [33]); (**b**) Deposit distributions of mercury in China in 2015 [34]; (**c**) Mercury consumption in China in 2015 [35] and (**d**) Distribution map of hazardous waste disposal companies in mainland China in the 12th Five-Year Plan period (data from the website of Department of Ecology and Environment of each province).

The UN Minamata Convention on Mercury, a global treaty, was signed in October 2013. It aims to control and reduce mercury emissions globally to reduce the damage caused by mercury to the environment and human health [30]. To reduce mercury emissions, the “Roadmap to Gradually Reduce the Mercury Content in Fluorescent Lamps in China” was promulgated by MIIT/MST/MEE (Ministry of Industry and Information Technology/Ministry of Science and Technology/Ministry of Ecology and Environment) of the China in February 2013 [28,31]. It was announced before the UN Minamata Convention on Mercury, which showed the advanced environmental awareness of the Chinese Government. Under their control, the industry pattern of FL production has also undergone obvious changes. Most provinces have cut down FL production and the FL market share in developed cities and provinces has decreased in mainland China (Figure 1a). Furthermore, China is one of countries having large mercury deposits. Most mercury deposits are distributed in some economically underdeveloped provinces, as shown in Figure 1b. Figure 1c shows that about 5% of total mercury production was used to produce lamps.

According to “Roadmap to Gradually Reduce the Mercury Content in Fluorescent Lamps in China”, the average mercury content of a single FL in 2015 decreased by about 80% compared with 2010 [28]. Due to the policy guidelines for reducing mercury and facilitating mercury immobilization, 60–70% of straight FLs were injected by nonvolatile and less harmful solid mercury in 2012, which means mercury consumption after 2015 was at least 92% less than that in 2010 [32]. However, the lamps containing mercury would not be eliminated in the next few years because of the imbalanced regional economic development among the provinces in China.

#### 3.1.2. SFL Collection

China is a populous country with a population of 1.39 billion [36], and the population is the key factor affecting the development of social economy. Of course, the population affects the output and recycling of SFLs too. According to the level and speed of economic development, China is divided into four regions, namely the Coastal, Central, Western and Northeastern regions with obvious regional economic gaps. Moreover, the regional economic gap among of rural areas, cities and ethnic autonomous areas in the four regions is more obvious.

Since the 1980s, the income gap in different regions of China has expanded rapidly, the gap in consumption level among residents in different regions has accelerated, and the consumption levels have tended to polarize, all of which may cause some adverse effects on FL (or SFL) management. First, low-consumption residents (LCRs) are used to purchasing cheap miscellaneous-brand FLs from retail stores. Generally, these FLs injected with excessive and uncontrolled amounts of liquid mercury are produced in small unknown workshops by hand. In addition, the quality of the filaments and glass tubes cannot reach the industry requirements, causing the emergence of more short-lived and breakable FLs. These poor-quality FLs directly lead to an increase in SFLs. Second, LCRs prefer to use high-brightness FLs with a minimum wattage. This has encouraged most FL manufacturers to inject excessive mercury into FL tubes to expand their sales market. Third, LCRs seldom pay for the proper household garbage treatment. They always dump SFLs into the wild by mixing them with household garbage, giving rise to serious environmental pollution in China [37]. Finally, LCRs usually break SFLs directly by hand to recycle the metal and plastic without any protection measures. Some junkmen even specialize in picking SFLs from dustbins and smashing them to recycle the aluminum and plastic.

**Figure 2:**
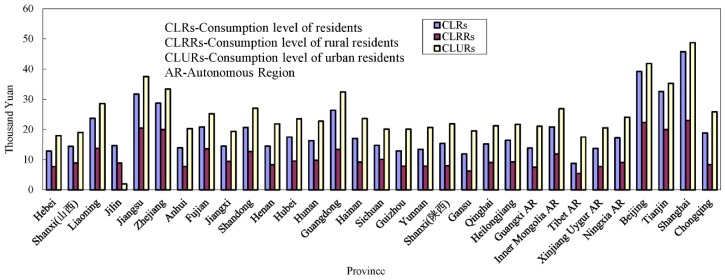
Consumption level of residents of mainland China in 2015 (7 yuan equals about $1 and data is from [38]).

In mainland China, most LCRs live in the rural areas of underdeveloped provinces (shown in Figure 2), where SFLs are always discarded into environment directly because few household waste disposal facilities have been built. Thus, it is important to recycle FLs through effective management of the manufacturer, sellers and purchaser of FLs. According to the same national standards for FLs production, the production capacity of FLs manufactures is different in every province, which is affected by the level of local economic development. Also, the effect of regulation on FLs manufactures is different because the FL production efficiency is different between economically developed and underdeveloped areas under the same tax revenue standard. On the other hand, the LCRs living in scattered rural areas and the urban periphery purchase FLs from small retailers or peddlers, which makes it easy for all them to disengage from regulation.

#### 3.1.3. SFL Treatment

The government is usually required to purchase FLs using a central procurement bidding method, and process SFLs through professional recycling companies. The financial section will not write off the cost if government units do not follow the above process. Households are the main FL consumer group, but contrary to the strict management on SFLs by the government, poor management of household SFLs always leads SFLs to be discarded randomly, for example, people living in rural areas are used to discarding SFLs into the environment and people living in urban areas always dump SFLs as household waste. Industrial and commercial customers are the second most important group after the household group. Some small industrial and commercial customers buy FLs, with a wide range of quality from poor to good, from retailers, wholesale markets or distributors, and throw all the SFLs away as municipal household wastes.

### 3.2. Comprehensive Evaluation

#### 3.2.1. Determination of Evaluation Factors

SFL management is a kind of public policy, which is influenced by economics, government, business, public and environment [39,40], so economics, government, business, public and environment are all determined as evaluation factors.

##### Strength Analysis of the Evaluation Factors

(a) Energy conservation and mercury emission from FLs and SFLs

Control of mercury during the producing and use of FLs and treatment of SFLs is important to coordinate the relationship between energy conservation and emission reduction, although the coordination is difficult and the control is influenced directly or indirectly by the consumption behaviors of residents. In fact, these consumption behaviors are influenced by some factors (e.g., Economy, Government, Business, Public and Environment) [41]. The difficulty value settings of these factors are as follows:

(1) Economy:

The economic development level influences people’s consumption behaviors a lot. Research has showed the absolute, relative and synthetic degrees of grey incidences of the quantity of economic growth and energy consumption were 0.87, 0.86, 0.86 in the period of 1999–2013, respectively, and 0.9 was the synthetic degrees of grey incidences of the quality of economic growth and energy consumption [42]. Another research showed that the absolute, relative and synthetic degrees of grey incidences of GDP and energy consumption were 0.62–0.94, 0.83–0.95, 0.73–0.93 in the different periods of 1985–2007, respectively [43]. These values indicate a high correlation between economy and energy consumption. That is to say, the economy has a high impact on energy conservation and emission reduction [44]. Therefore, it is reasonable that 0.9 was determined as the high correlation value;

(2) Government:

Government restrictedly influences consumption behaviors by providing guidance [45]. Then the high correlation value was set to 0.2;

(3) Business:

Businesses always pay attention to their own practical benefits and influence consumption behaviors through advertisements and market expansion, but this is only efficient within a certain range [46]. Its influence is less than 50% and the high correlation value was set to 0.4;

(4) Public:

Except for the direct energy consumption impact of the household sector, people’s consumption behaviors indirectly affect the energy consumption of multiple production sectors [47]. Some manufacturers of FLs increase the amount of mercury added to fluorescent tubes to cater to consumer preferences. Then the high correlation value was set to 0.8;

(5) Environment:

The environment is always polluted by consumption production. Although environmental degradation may have a direct impact on residents’ economic growth and consumption behaviors, its feedback on environmental pollution lags behind [48]. Therefore, the high correlation value was set to 0.4, which is the same as that of business.

(b) SFL collection

It is necessary to collect SFLs separately from household solid waste. However, the separate collection of SFLs is remarkably influenced by the behaviors of others, facility conditions and moral obligations. Meanwhile, resident’s willingness to pay for separate collection is affected by ages, perceptions of results and government policies [49]. The experiences of developing areas in China show that the government should supply recycling facilities and enforce laws to promote solid waste collection, business can take part in the collection action by financial support, and residents’ behaviors, attitudes and willingness are the key factors for collection [50]. Tian et al.’s survey showed that 68.6% of Beijing residents were willing to pay extra fees to safely dispose of scrap fluorescent lamps [51]. This indicates that public attitudes of residents who have learned about environmental problems have a greater impact on collection than other factors [52,53]. According to the above messages, the high correlation values of the five factors (Economy, Government, Business, Public and Environment) can be set to 0.5, 0.4, 0.2, 0.8, and 0.8, respectively.

(c) SFL treatment

The implementation of SFL treatment is mainly undertaken by professional recycling enterprises, whose production, management and operation are not interfered with the government. The business has nothing to do with the enterprise’s behavior because of its particularity. Among the five factors of economy, government, business, public and environment, there are three factors influencing SFL treatment, namely economy, public and environment. SFL treatment is a public welfare behavior with less economic influence, so according to the difficulty value setting method of “Energy conservation and mercury emission from FLs and SFLs”, the high correlation value of the five factors can be set to 0.6, 0.5, 0.5, 0.7, and 0.4, respectively. Detailed results of the membership of the factors are listed in Table 1.

##### Determination of Weight

Although the factors that affect the environment are complex, public perceptions and preferences cannot be ignored. Public, economy, government, business and environment affect social behavior to varying degrees according to reference [54]. Then the weight was set as follows:

$$\begin{array}{ll} A & =\left\{\;\mathrm{Public},\;\mathrm{Economy},\;\mathrm{Government},\;\mathrm{Business},\;\mathrm{Environment}\right\}\\ & =\left\{A_{1},\;A_{2},\;A_{3},\;A_{4},\;A_{5}\right\}=\left\{0.3,\;0.25,\;0.20,\;0.15,\;0.10\right\} \end{array}$$

#### 3.2.2. Evaluation

(1) Evaluation object set

$$\begin{array}{ll} B & =\left\{\;\mathrm{Energy}\;\mathrm{conservation}\;\mathrm{and}\;\mathrm{mercury}\;\mathrm{emission}\;\mathrm{from}\;\mathrm{FLs}\;\mathrm{and}\;\mathrm{SFLs},\;\mathrm{FLs}\;\mathrm{collection},\;\mathrm{FLs}\;\mathrm{treatment}\right\}\\ & =\left\{B_{1},\;B_{2},\;B_{3}\right\} \end{array}$$

(2) Evaluation matrix

$$B_{1}=\left\{\begin{array}{cc} 0.8 & 0.2\\ 0.9 & 0.1\\ 0.2 & 0.8\\ 0.4 & 0.6\\ 0.4 & 0.6 \end{array}\right\}\;B_{2}=\left\{\begin{array}{cc} 0.8 & 0.2\\ 0.5 & 0.5\\ 0.4 & 0.6\\ 0.2 & 0.8\\ 0.8 & 0.2 \end{array}\right\}\;B_{3}=\left\{\begin{array}{cc} 0.7 & 0.3\\ 0.6 & 0.4\\ 0.5 & 0.5\\ 0.5 & 0.5\\ 0.4 & 0.6 \end{array}\right\}$$

Here Fuzzy Composite Operators employ an ordinary matrix product algorithm. Then:

$$V=A\cdot B\Rightarrow \{\begin{array}{l} V_{1}=A\cdot B_{1}=\left\{0.605,\;0.395\right\}\\ V_{2}=A\cdot B_{2}=\left\{0.555,\;0.445\right\}\\ V_{3}=A\cdot B_{3}=\left\{0.575,\;0.425\right\} \end{array}$$

(3) Evaluation analysis

According to the calculation, membership degree of difficulty can be ranked as:

$$B_{1}>B_{3}>B_{2}\Leftrightarrow 0.605>0.575>0.555$$

The calculation indicates that “energy conservation and mercury emission from FLs and SFLs” is the biggest one of the three problems.

### 3.3. Solutions to Control the Mercury Pollution from FLs and SFLs

According to the above analysis, mainland China should adopt the following methods and measures to control mercury pollution:

- Establishing a recycling system for SFLs
- Improving the disposal capacity of mercury
- Improving the disposal technology of mercury
- Strengthening the management of mercury
- Developing the LED industry

#### 3.3.1. Establishing a Recycling System for SFLs

Solid wastes are large sources of pollution that contain all kinds of organic and inorganic pollutants. Among solid wastes, municipal solid wastes (MSWs) are difficult to classify, especially hazardous wastes. There are many kinds of hazardous wastes in MSWs, such as SFLs, mercury thermometers, used batteries and expired drugs. If the recycling system of SFLs can be established as a demonstration, the other hazardous wastes in the MSWs will be also well sorted and recycled, effectively reducing the pollution coming from households. However, it is not easy to establish recycling systems for SFLs, because most SFLs come from households, and the disperse and independent nature of residents makes their behaviors uncontrollable. Therefore, the collection and sorting are two key steps that are difficult to realize.

Establishment of a recycling system for SFLs is a big problem that the government of China has to face, and it means a lot. In 2014 the city of Chengdu in Sichuan Province started the “China Fluorescent Lamp Collection and Treatment Demonstration Project” (FLCTDP) that was a sub-project of “The EU-China Environmental Sustainability Programme” (ESP) funded by the European Commission. Four sites of Chengdu city were selected as the SFL collection demonstrations of China, with the goal of achieving a SFL collection rate of 60% [55]. The project will provide valuable experience for the whole country.

#### 3.3.2. Improving the Disposal Capacity of Mercury

As an important heavy metal pollutant, mercury use has been reduced in industrial and commercial applications in the past years, but other kind of hazardous wastes containing mercury (such as SFLs) have increased rapidly and the disposal rate is low. Despite the large numbers of hazardous waste disposal companies in mainland China (Figure 1d), they cannot meet the actual requirements of hazardous waste disposal. Environmental statistics showed that there were about 45.74 million tons of hazardous wastes produced, of which 39.4 thousand were discarded without any disposal. There were only 159 hazardous waste treatment companies and 184 medical waste disposal companies in mainland China. There are even no hazardous waste treatment companies in some administrative regions (e.g., Shanxi Province, Henan Province, Guizhou Province, Qinghai Province, Chongqing city, Inner Mongolia Autonomous Region, Tibet Autonomous Region, Ningxia Autonomous Region and Xinjiang Uygur Autonomous Region) by 2007 [56]. Until 2012, there were only 11 special companies with the capability to dispose of hazardous wastes containing mercury (HWCM), of which merely four could handle SFLs [57].

To deal with mercury, the MEE evaluated the disposal technology, management and economic policy of mercury in developed countries, learned management system as well as countermeasures and measures to promote developing pollution prevention and disposal technique of mercury, and subsequently delivered “Guidelines on Available Technologies of Pollution Prevention for Mercury-containing Waste Disposal” (GATPPMCWD, Draft for Comment) in 2014 [58]. The GATPPMCWD surveyed the existing disposal technology and related policy in mainland China, found the weaknesses of the feasible technology to treat with mercury, conducted meticulous study on the Basel Convention and the Minamata Convention on Mercury, and referenced some better practical experience and available technology of some countries (e.g., USA, EU and Japan).

The introduction of GATPPMCWD indicates that the government of China is focused on the special management of mercury disposal. The systems of technology and management will be more perfect and effective in mainland China if more other special management of mercury disposal is focused on GATPPMCWD or similar programs. In the fact, with the Chinese Government’s emphasis on mercury management, more and more qualified enterprises have been permitted to engage in business operations involving mercury, which provides conditions for the expansion of mercury disposal facilities.

#### 3.3.3. Improving the Disposal Technology of Mercury

Unfortunately, most landfills have no special facilities for SFL pre-treatment. SFLs emit mercury into the atmosphere after being destroyed in the decompression and compaction process of municipal household wastes in the atmosphere. There are three ways to dispose of mercury in mainland China: the first is safe landfill with MSWs, the second is incineration with MSWs, and the third is recycling [59,60,61]. Regarding safe landfill and incineration, the MEE has published “General specifications of engineering and technology for hazardous waste disposal” in 2014 [62], but there were few guidance documents on mercury recycling. Now more engineering and technology are needed to meet the demand for mercury disposal and recycling in mainland China. However, the facilities and techniques are still weak. With the development of industries and the improvement of environmental protection requirements, it is urgent to establish more mercury disposal facilities in mainland China, and it is necessary to encourage more special companies to engage in mercury disposal.

#### 3.3.4. Strengthening the Management of Mercury

The government of China has revised “Integrated emission standard of air pollutants” (IESAP) several times to reduce particulates emissions from manufactures [63], and in 2012, the MEE and General Administration of Quality Supervision, Inspection and Quarantine (GAQSIQ) announced the “Ambient air quality standard” (AAQS) [64]. The standards added limits to the contents of particulate PM_2.5_ and PM_10_. These can not only meet the terms of the UN Minamata Convention on Mercury, but also reduce heavy metal pollution in China.

**Figure 3:**
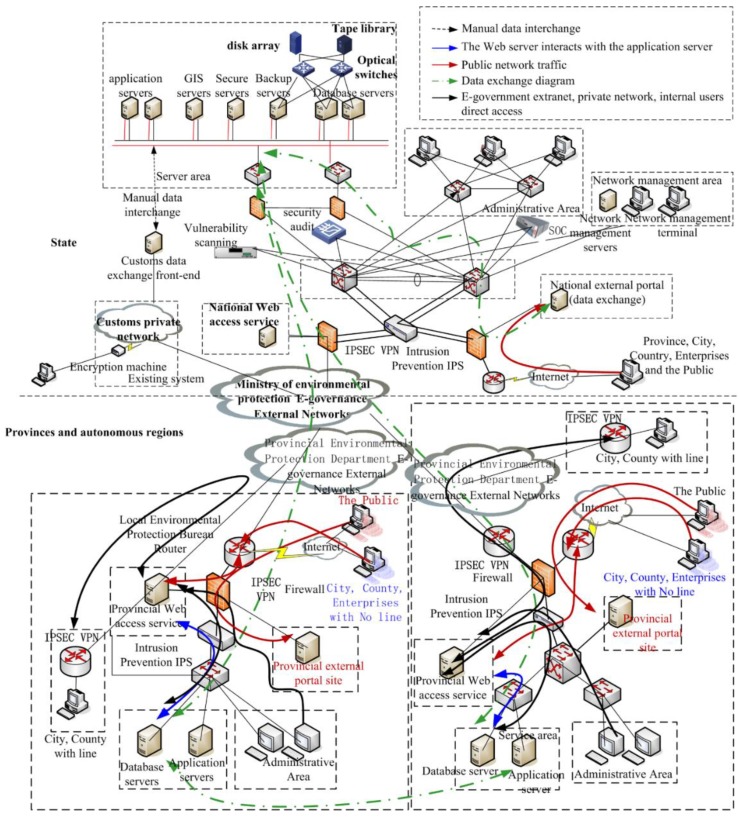
Diagram of electronic waste information management network [65].

In mainland China, the management of spent lamps is the most difficult aspect because most spent lamps come from households, although many successful management experiments on solid wastes can be referenced by the management on heavy metals. For example, the E-wastes management system has been set based on the electronic waste information management network in China, as shown in Figure 3. In this system, business portals for environmental protection departments at all levels were constructed, a business application system for the implementation of the actual electrical and electronic waste products processing and data input, data collection and data report operating functions was built, and the supervision and inspection functions of enterprises for processing electrical and electronic waste products were established. All of these have greatly improved the efficiency of E-wastes management.

#### 3.3.5. Developing the LED Industry

The best way to avoid heavy metal pollution is to take replace heavy metals with eco-friendly materials. In 2012, the MIIT announced the catalog of “Encouraged Substitutes to Toxic and Hazardous Raw Materials” (2012 draft) and published it again in 2016 catalog (2016 draft) [66,67]. Many hazardous materials listed in the catalog were replaced. According to aforementioned comprehensive evaluation, the biggest problem of FLs that mainland China is facing is “Energy conservation and mercury emission from FLs and SFLs”. Under the UN Minamata Convention on Mercury for the green lighting industry, the solution to save energy and avoid mercury pollution from SFLs during collection and disposal is that LED lamps were promoted to take the place of FLs. That is the opportunity that mainland China is facing.

Although the content of gallium arsenide (GaAs) in LED lamps was not allowed exceed 0.1% of the quality of the LED component [68], so far, Chinese Government has not put LEDs-related items in the National Hazardous Waste List [69]. Considering the energy efficiency and environmental compatibility, LEDs should replace FLs. With the progress in manufacturing processes and design technologies, the price of LEDs is declining. The excellent soft light of LEDs makes LED light become excellent area lights, which can eliminate glare and visual fatigue, and improve visual effects. LEDs have been accepted and favored by more and more consumers.

In 2015, China’s LED packaging output share reached 21%, ranking first in the world. By 2016, the packaging enterprises in China have provided more than 70% of the packaging output worldwide. In the next few years, China’s LED packaging industry output value will maintain a 13–15% growth rate and it is expected that the scale of China’s LED packaging will further increase to $19.2 billion in 2020 [70]. In the next 10 years, spent LED lamps will surely account for a large part of solid wastes. Then, arsenic, rare (such as gallium, indium) [71], heavy (e.g., nickel, lead) and precious (such as silver) [72] metals must be recycled to keep sustainable development and gain benefits, so now is the time for our government to consider the future management of LED lamp recycling.

## 4. Conclusions

In summary, the pollution problems caused by mercury from fluorescent lamps and spent fluorescent lamps were discussed. The reasons for this pollution were analyzed and the influence of the processing of fluorescent lamps and the collection and disposal of spent fluorescent lamps were evaluated based on fuzzy theory. Results show that “energy conservation and mercury emission from fluorescent lamps and spent fluorescent lamps” is the biggest problem that causes mercury pollution. The best way to solve these problems is by replacing fluorescent lamps with energy-saving and environment-friendly LEDs in mainland China. The development process of China’s lighting industry is like that of other countries. Consequently, the experience of China can also be used by other countries for reference, especially developing and undeveloped countries.

## Figures and Tables

**Table 1:** Membership of factors.

Factor	The Difficulty between Energy Conservation and Mercury Emission		The Difficulty of SFL Collection		The Difficulty of SFL Treatment	
Membership	High	Low	High	Low	High	Low
Public	0.8	0.2	0.8	0.2	0.7	0.3
Economy	0.9	0.1	0.5	0.5	0.6	0.4
Government	0.2	0.8	0.4	0.6	0.5	0.5
Business	0.4	0.6	0.2	0.8	0.5	0.5
Environment	0.4	0.6	0.8	0.2	0.4	0.6
